# Analysis and Identification of Genes Associated with the Desiccation Sensitivity of *Panax notoginseng* Seeds

**DOI:** 10.3390/plants12223881

**Published:** 2023-11-17

**Authors:** Yanan Wang, Weiqing Wang, Xiulian Chi, Meng Cheng, Tielin Wang, Xiaori Zhan, Yunjun Bai, Chenjia Shen, Xiaolin Li

**Affiliations:** 1 State Key Laboratory for Quality Ensurance and Sustainable Use of Dao-Di Herbs, National Resource Center for Chinese Materia Medica, China Academy of Chinese Medical Sciences, Beijing 100700, China; wangyn@nrc.ac.cn (Y.W.); xiulian68@126.com (X.C.); mengzai@163.com (M.C.); wtl82@163.com (T.W.); baiyunjun@126.com (Y.B.); 2 Chinese Materia Medica, China Academy of Chinese Medical Sciences, Beijing 100700, China; wwq0814@ibcas.ac.cn; 3 Zhejiang Provincial Key Laboratory for Genetic Improvement and Quality Control of Medicinal Plants, Hangzhou Normal University, Hangzhou 310036, China; cortex@163.com (X.Z.); shencj@hznu.edu.cn (C.S.)

**Keywords:** desiccation sensitivity, differentially expressed gene, *P. notoginseng*, recalcitrant seed

## Abstract

*Panax notoginseng* (Burk.) F. H. Chen, a species of the genus Panax, radix has been traditionally used to deal with various hematological diseases and cardiovascular diseases since ancient times in East Asia. *P. notoginseng* produces recalcitrant seeds which are sensitive to desiccation and difficult to store for a long time. However, few data are available on the mechanism of the desiccation sensitivity of *P. notoginseng* seeds. To gain a comprehensive perspective of the genes associated with desiccation sensitivity, cDNA libraries from seeds under control and desiccation processes were prepared independently for Illumina sequencing. The data generated a total of 70,189,896 reads that were integrated and assembled into 55,097 unigenes with a mean length of 783 bp. In total, 12,025 differentially expressed genes (DEGs) were identified during the desiccation process. Among these DEGs, a number of central metabolism, hormonal network-, fatty acid-, and ascorbate-glutathione-related genes were included. Our data provide a comprehensive resource for identifying the genes associated with the desiccation sensitivity of *P. notoginseng* seeds.

## 1. Introduction

For higher plants, seed formation implies the beginning of a new life cycle. Several important features, including development, desiccation sensitivity, vigor, and germination, are involved in the seed maturation process [1]. Robert grouped seeds into orthodox and recalcitrant seeds according to their storage properties [2]. Orthodox seeds produce desiccation tolerance at late developmental stages [3]. Due to desiccation tolerance, the orthodox seeds can be stored for a long period of time under extreme environmental or artificial conditions [4]. In contrast, recalcitrant seeds are desiccation-sensitive and cannot survive during long-time conservation [5,6]. This storage limitation causes a severe problem for germplasm resource conservation and agricultural production practice [4]. Thus, it is important to understand the mechanisms of desiccation sensitivity underlying recalcitrant seeds.

Several protective mechanisms have been proposed to explain seed desiccation, such as the accumulation of dehydrins and late embryogenesis abundant (LEA) proteins, the efficient regulation of the antioxidant system, the inhibition of metabolism, and the presence of a repair system during the rehydration process [7,8]. The accumulation of reactive oxygen species (ROS) is considered to be a major reason for the damage caused by dehydration [9]. Desiccation sensitivity may be associated with ROS production in the late development stage, and a more effective operation of the antioxidant system ensures the structural and functional integrity of mitochondria in seeds during the desiccation process [3]. Furthermore, alterations in metabolic pathways, such as sugar metabolism, lipid metabolism, and dehydrins metabolism, have been reported to contribute to the desiccation sensitivity of various plant species [10,11]. Seeking a better understanding of seed desiccation sensitivity has become a pertinent region of research recently.

*Panax notoginseng* (Burk.) F. H. Chen, a member of the *Araliaceae* family, is a slow-growing herb and has been used in traditional Chinese medicine for a long time [12]. In the past years, many important chemical and pharmacological compounds, including flavonoids, saponins, amino acids, polysaccharides, and fatty acids, have been isolated from *P. notoginseng* [13]. Triterpenoid saponins are thought to be a major pharmacological ingredients, and many different types of triterpenoid saponins have been identified [14]. Freshly matured *P. notoginseng* seeds have small, underdeveloped embryos surrounded by abundant endosperm and only can germinate after experiencing an after-ripening process [15]. So, they have morphophysiological dormancy (MPD) [14]. Previous studies showed that exogenous gibberellic acid could shorten the after-ripening process, and abscisic acid could prolong the dormancy of the seed of *P. notoginseng* [16,17]. However, *P. notoginseng* seeds also have recalcitrant traits, such as not undergoing maturation drying, a high water content, high sensitivity to dehydration, and a short life span in natural conditions [15,18]. Hence, understanding the regulation mechanism of *P. notoginseng* seed recalcitrance, and accordingly developing conservation practices, has practical and scientific importance.

Transcriptome sequencing is a highly effective approach to analyze gene transcripts in non-model plants. In our study, genome-wide transcriptome profiling was carried out to identify the genes associated with the desiccation sensitivity of *P. notoginseng* seed embryos. A large number of differentially expressed genes (DEGs) were identified during the desiccation process of *P. notoginseng* embryos containing glycolysis, TCA cycle, LEA proteins, lipids, and ROS. This information will give us an opportunity to understand the molecular mechanisms and regulatory networks of desiccation tolerance in recalcitrant seeds such as *P. notoginseng*, determine and regulate the key factors involved in *P. notoginseng* seeds storage, and improve the genetic information of recalcitrant *P. notoginseng*. This study could help to improve the desiccation tolerance and prolong the storage life of *P. notoginseng* seeds.

## 2. Results

### 2.1. Changes in Water Contents and Survival Ratios during Dehydration Process

**Figure 1:**
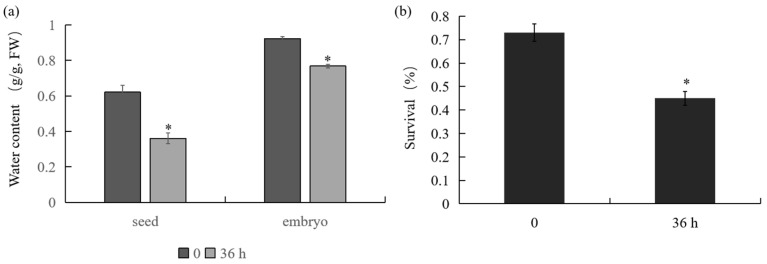
Changes in water contents and survival ratios during dehydration process. (**a**) Water contents of *P. notoginseng* seeds and embryos at 0 and 36 h after dehydration treatment. (**b**) Survival ratios of *P. notoginseng* seeds at 0 and 36 h after dehydration treatment. Significant differences in the water contents and survival ratios between 0 and 36 h were indicated by “*”.

In our study, the water contents and survival ratios of *P. notoginseng* seeds and embryos during the dehydration process were measured. The water contents of *P. notoginseng* seeds and embryos at 0 h were 0.62 (g·g^−1^, FW) and 0.92 (g·g^−1^, FW), respectively. After 36 h dehydration, the water content decreased to 0.36 (g·g^−1^, FW) and 0.77 (g·g^−1^, FW) (Figure 1a). The survival ratio of seeds at 0 h was 73% and was reduced to 45% after 36 h of dehydration (Figure 1b).

### 2.2. Sequencing, Assembly, and Functional Annotation

To obtain a reference transcriptome of *P. notoginseng*, six independent cDNA libraries were combined to obtain a comprehensive survey of the transcripts. Pair-wise Pearson’s correlation coefficients of all six samples (three replicates × two stages) showed a high repeatability of this experiment (Figure S1a). Raw reads from six complementary DNA libraries were qualified and adapter-removed to produce a total of 46,581,204 clean reads comprising 69.88 Gb of sequence data (Table S1). Then, all the clean reads from two dehydration stages were assembled, resulting in 285,646 transcripts (N50: 1756) with a mean length of 994 bp. For each dataset, approximately 57.9% of reads could be mapped to the reference transcriptome. Clustering of the transcripts resulted in 165,472 unigenes (N50: 1086) with a mean length of 681 bp (Figure S1b,c).

To functionally annotate the unigenes of *P. notoginseng*, their sequences were searched against several protein databases. There were 65,849 unigenes (39.79%) that could be annotated in the non-redundant protein sequence (Nr) database, 43,754 (26.44%) in the nucleotide sequence (Nt) database, 24,125 (14.57%) in the Kyoto Encyclopedia of Genes and Genomes (KEGG) database, 46,874 (28.32%) in the SwissProt database, 42,740 (28.32%) in the PFAM database, 44,187 (26.7%) in the Gene Ontology (GO) database, and 22,467 (13.57%) in the KOG database (Figure S1d).

### 2.3. GO and KEGG Classifications of Unigenes

**Figure 2:**
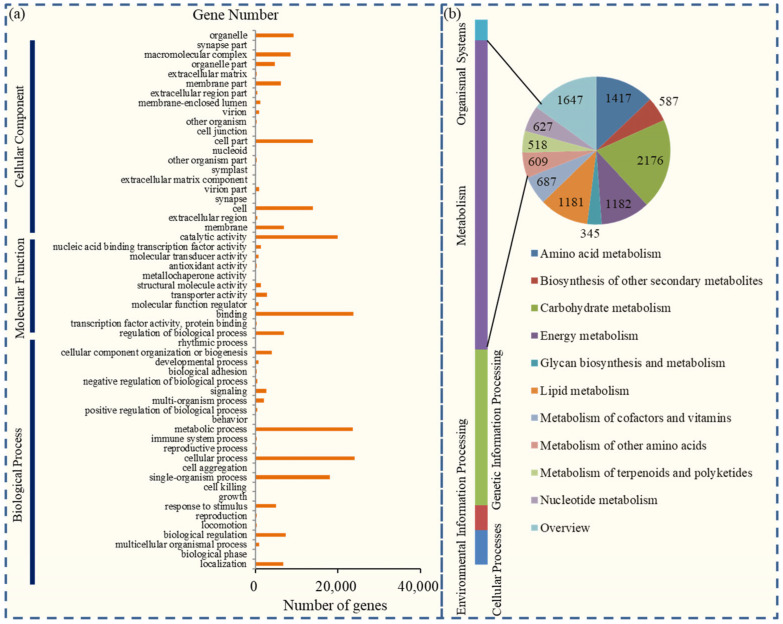
Classification of enriched GO and KEGG terms in the unigenes of *P. notoginseng*. (**a**) Most unigenes could be assigned to 56 functional terms that belonged to three major GO categories, biological process, cellular component, and molecular function. (**b**) In total, 24,125 unigenes from *P. notoginseng* were assigned to 283 signaling and metabolic pathways, including pathways related to cellular process, environmental information processing, genetic information processing, metabolism, and organismal systems.

In *P. notoginseng*, most of the unigenes could be assigned to 56 functional terms that belonged to three major GO categories, including “molecular function”, “cellular component”, and “biological process” (Table S2). For biological process, “cellular processes” (24,113 unigenes), “metabolic processes” (23,572 unigenes), and “single organism process” (18,005 unigenes) were dominant GO terms; for cellular component, the major GO terms were “cell” (13,948 unigenes), “cell part” (13,941 unigenes), and “organelle” (9263 unigenes); and for molecular function, most of the unigenes were related to “binding” (23,743 unigenes), “catalytic activity” (19,938 unigenes), and “transporter activity” (2766 unigenes) (Figure 2a).

Most unigenes could be mapped onto the reference metabolic pathways in the KEGG database. In total, 24,125 unigenes from *P. notoginseng* were assigned to 283 signaling and metabolic pathways, including pathways related to cellular process, environmental information processing, genetic information processing, metabolism, and organismal systems (Table S3). The most enriched KEGG pathways were carbohydrate metabolism (2176 unigenes), amino acid metabolism (1417 unigenes), energy metabolism (1182 unigenes), and lipid metabolism (1181 unigenes) (Figure 2b).

### 2.4. Transcriptional Changes Associated with Desiccation Sensitivity

**Figure 3:**
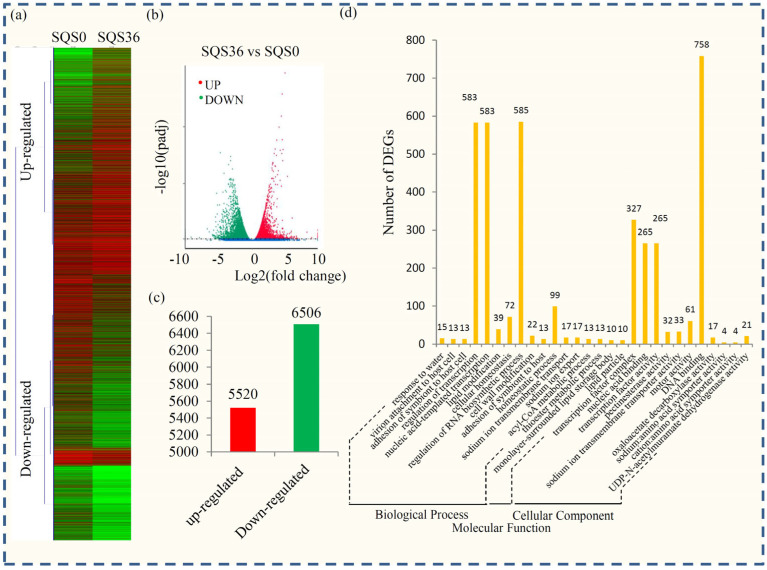
Transcriptional variation in *P. notoginseng* embryos during desiccation process. (**a**) Expression profiles of the differential expressed unigenes during desiccation process in *P. notoginseng* were shown by a heatmap. Red indicates up-regulated genes and blue indicates down-regulated genes. (**b**) Significance analysis of all DEGs between normal and malformed flowers by a volcano plot. (**c**) The numbers of up-regulated genes and down-regulated genes at SQS36 compared to SQS0. (**d**) GO enrichment analysis of DEGs between SQS36 and SQS0.

Transcriptional changes in responses to desiccation were analyzed by comparing the transcriptomes from the control embryos (SQS0) with the seeds under the 36 h desiccation treatment (SQS36). The global gene expression profiles showed the differences in the expression levels between SQS0 and SQS36 (Figure 3a). In total, 12,025 genes, including 5520 up- and 6505 down-regulated genes, were identified as DEGs (Table S4, Figure 3b,c).

The representative GO terms were analyzed to obtain useful information about the DEGs. Enrichment analysis indicated that 28 GO terms referring to different biological processes, such as “DNA binding transcription factor”, “regulation of transcription”, “nucleic acid-templated transcription”, “regulation of RNA biosynthetic process”, and “transcription factor complex”, were significantly enriched in the DEGs (Figure 3d and Table S5). In the KEGG classification, a large number of DEGs were classified into 310 KEGG pathways, among which 16 KEGG terms were significantly enriched (Table S6). In these enriched terms, “Plant hormone signal transduction” (ko04075), “cell cycle” (ko04110), “Phagosome” (ko04145), “Fructose and mannose metabolism” (ko00051), and “DNA replication” (ko03030) were the top five largest KEGG terms.

### 2.5. Protein–Protein Interaction (PPI) Network Analysis of DEGs

To explore the biological processes involved in the *P. notoginseng* seed desiccation responsive genes, the PPIs among the DEGs were analyzed. The PPI network for the *P. notoginseng* desiccation process had a number of proteins connected by a series of direct physical interactions. A comprehensive PPI network was constructed, and different enriched interaction clusters were indicated by different cycles (Figure S2). Interestingly, the proteins associated with glycolysis showed significant differences in the comparison between SQS0 and SQS36.

### 2.6. Identification of Glycolysis- and TCA Cycle-Related DEGs

**Figure 4:**
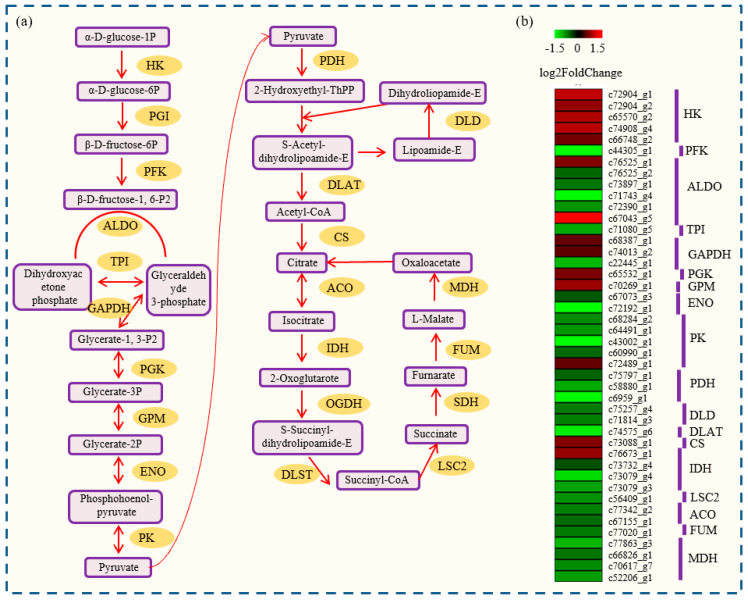
Transcript abundance changes in central metabolism-related genes. (**a**) Overview of the central metabolism in *P. notoginseng*. Enzyme abbreviations are: HK: hexokinase; PGI: phosphoglycerate isomerase; PFK: 6-phosphofructokinase 1; ALDO: fructose-bisphosphate aldolase; TPI: triosephosphate isomerase; GAPDH: glyceraldehyde 3-phosphate dehydrogenase; PGK: phosphoglycerate kinase; GPM: probable phosphoglycerate mutase; ENO: enolase; PK: pyruvate kinase; PDH: pyruvate dehydrogenase; DLD: dihydrolipoamide dehydrogenase; DLAT: pyruvate dehydrogenase; CS: citrate synthase; ACO: aconitase; IDH: isocitrate dehydrogenase; OGDH: ketoglutarate dehydrogenase; LST: succinyl-CoA synthase; SDH: succinate dehydrogenase; FUM: fumarase; MDH: malate dehydrogenase. (**b**) Expression changes in the genes associated with central metabolism-related genes. Red indicates up-regulated genes and green indicates down-regulated genes.

A large number of DEGs in *P. notoginseng* showed homology to known glycolysis- and TCA cycle-related genes in the National Center for Biotechnology Information (NCBI) database. In total, 49 glycolysis and 24 TCA cycle-related DEGs were identified. Several glycolysis-related genes were up-regulated during the seed desiccation process. For example, *five hexokinase (HK)* genes, one *phosphoglycerate kinase (PGK)* gene, and one *probable phosphoglycerate mutase (GPM)* gene were up-regulated at SQS36 compared with SQS0. Interestingly, most of the TCA cycle-related genes, except for one *pyruvate kinase (PK)* gene, one *citrate synthase (CS)* gene, and *isocitrate dehydrogenase (IDH)* gene, were down-regulated during the seed desiccation process (Figure 4).

### 2.7. Identification of Hormone-Related DEGs

**Figure 5:**
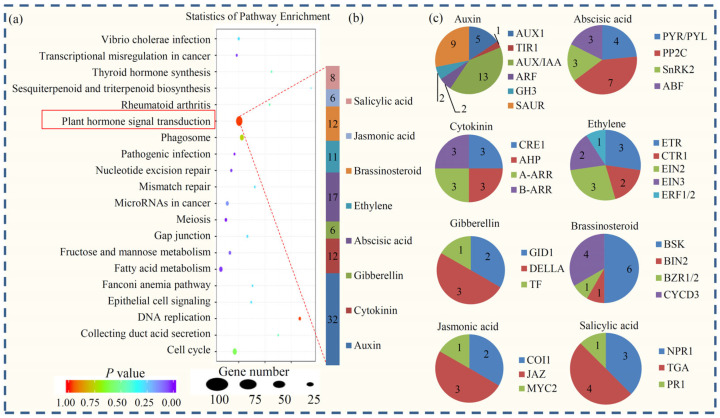
Transcript abundance changes in hormone-related genes. (**a**) Pathway enrichment analysis of DEGs. (**b**) The numbers of genes involved in different hormone signaling pathways. (**c**) The number of genes belonging to each component of various hormone signaling pathways.

Hormones are involved in seed development, desiccation sensitivity, and germination [19]. In our study, KEGG enrichment analysis showed that the “Plant hormone signal transduction” pathway consisted of the largest proportion of DEGs (Figure 5a). The number of DEGs belonged to each hormonal signaling pathway in *P. notoginseng* is shown in Figure 5b.

Interestingly, the largest number of hormone-related DEGs belonged to the auxin signaling pathway. For auxin homeostasis, two *gretchen hagen three (GH3)* genes were identified as DEGs; for auxin transport, five *influx carrier* (*AUX*) genes were identified as DEGs; and for auxin downstream response, two *auxin response factor (ARF)* genes, thirteen *Aux/3-Indoleacetic acid (IAA)* genes, and nine *small auxin-up RNA (SAUR)* genes were identified as DEGs. Furthermore, 12 DEGs, including 3 *cytokinin receptor (CRE1)* genes, 3 *arabidopsis histidine phosphotransfer proteins (AHP)* genes, 3 *type-A Arabidopsis response regulators (A-ARR)* genes, and 3 *type-B Arabidopsis response regulators (B-ARR)* genes, were identified in the cytokinin pathway. For the gibberellin pathway, two *gibberellin insensitive dwarf1 (GID1)* receptor genes, three *DELLA* genes, and one *transferrin (TF)* gene were identified as DEGs. For the abscisic acid pathway, four *PYR/PYL* genes, seven *protein phosphatase 2C (PP2C)* genes, three *sucrose non-fermenting 1-related protein kinase 2 (SnRK2)* genes, and three *antibacterial factor (ABF)* genes were identified as DEGs. For the ethylene pathway, three *arabidopsis ethylene receptor (ETR)* genes, two *constitutive triple-response 1 (CTR1)* genes, three *ethylene insensitive 2 (EIN2)* genes, three *ethylene insensitive 3 (EIN3)* genes, and one *ethylene response factor 2 (ERF2)* gene were identified. For the brassinosteroid pathway, *brassinosteroid-signaling kinase (BSK)* genes, one *brassinosteroid insensitive 2(BIN2)* gene, one *brassinazole resistant transcription factor 1/2 (BZR1/2)* gene, and four *cell cycle genes D-type cyclin 3 (CYCD3)* genes were identified as DEGs. For the jasmonic acid pathway, two *coronatine-insensitive 1 (COI1)* genes, three *jasmonate ZIM domain (JAZ)* genes, and one *myelocytomatosis protein 2 (MYC2)* gene were identified. Lastly, three *nonexpressor of pathogenesis-related genes 1 (NPR1)* genes, four *TGACG motif binding factor (TGA)* genes, and one *PR1* gene were identified in the salicylic acid pathway (Figure 5c).

The role of ABA in the regulation of desiccation tolerance in germinated *Arabidopsis* has been well studied [20]. The expression of 17 ABA signaling genes was determined during the desiccation process in *P. notoginseng* (Table S7). Interestingly, four *PYR/PYL* genes and three *SnRK2* genes were reduced significantly at SQS36 compared with SQS0, and three *ABF* genes were induced significantly at SQS36 compared with SQS0.

### 2.8. Expression Analysis of Fatty Acid-Related Unigene in P. notoginseng during Seed Desiccation Process

**Figure 6:**
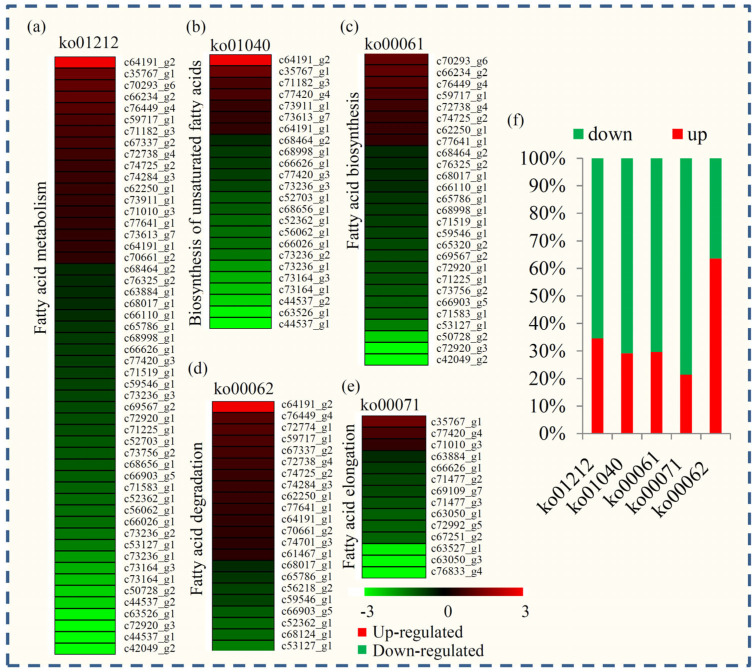
Identification and expression analysis of the KEGG terms related to fatty acid metabolism. (**a**) Differential expression profiling of “fatty acid metabolism”-related genes. (**b**) “Biosynthesis of unsaturated fatty acids”-related genes. (**c**) “Fatty acid biosynthesis”-related genes. (**d**) “Fatty acid degradation”-related genes. (**e**) “Fatty acid elongation”-related genes. (**f**) The proportion of up- and down-regulated genes in each “fatty acid”-related KEGG term. Red indicated the up-regulated genes and green indicated the down-regulated genes.

Based on the GO annotation, five fatty acid-related KEGG terms, including “Fatty acid metabolism” (ko01212), “Fatty acid degradation” (ko00071), “Biosynthesis of unsaturated fatty acids” (ko01040), “Fatty acid biosynthesis” (ko00061), and “Fatty acid elongation” (ko00062), were identified. The expression pattern of the genes from fatty acid-related KEGG terms is shown in Figure 6 and Table S8. Many fatty acid-related genes were down-regulated during the seed germination process. For example, the expression of about 80% of genes associated with fatty acid degradation reduced at SQS36 compared with SQS0. The expression level of nearly 70% of genes related to fatty acid biosynthesis and unsaturated fatty acids biosynthesis was down-regulated at SQS36 compared with SQS0.

### 2.9. Identification of LEA Protein Encoding Genes

LEA proteins are abundant in recalcitrant and orthodox legume seeds during the desiccation process [21]. A number of LEA-encoding genes were identified (Table S9). Among these genes, 14 *LEA* genes, including 5 down-regulated and 9 up-regulated genes, showed significant changes at SQS36 compared with SQS0.

### 2.10. Identification of Genes Associated with Removal of ROS

Most of the genes associated with the removal of ROS could be classed into three major categories, including thiol-dependent antioxidant proteins, the aldehyde dehydrogenase-related cycle, and the ascorbate-glutathione cycle [22]. In our study, one *thioredoxin peroxidase* gene was identified as a DEG, and it was down-regulated during the seed desiccation process; seven *alcohol dehydrogenase (ADH)* genes were identified as DEGs, including one up-regulated and six down-regulated genes, during the seed desiccation process; and four genes encoding three enzymes involved in the glutathione-ascorbate cycle were identified as DEGs during the seed desiccation process. For the ascorbate-glutathione cycle, one *monodehydroasorbate reductase (MDHAR)* gene, two *dehydroasorbate reductase (DHAR)* genes, and one *glutathione reductase (GR)* gene were identified as DEGs in the seed desiccation process (Table 1).

### 2.11. Validation of RNA-Seq Results Using qPCR

**Figure 7:**
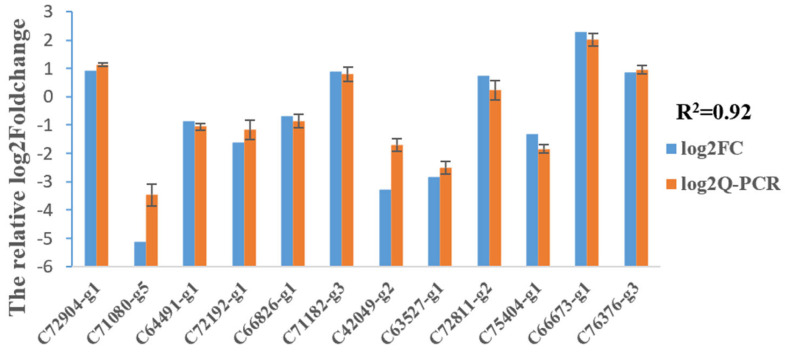
Validation using qPCR. Comparison of the relative log2 (fold changes) between RNA-seq and qPCR compared to the control, as normalized to expression of the 18S rRNA gene.

To verify the accuracy of the RNA-seq data, twelve genes were chosen for validation using qPCR. As shown in Figure 7, the correlation coefficient of the relative log2 (fold changes) was 0.92 between RNA-seq and qPCR, suggesting the correctness of the bioinformatics analysis for the transcriptomic sequencing data.

## 3. Discussion

*P. notoginseng* is an important Chinese medicinal plant with a number of pharmacologically effective components [23]. A variety of *P. notoginseng*-specific secondary metabolites, such as ginsenosides and gypenosides, have been isolated and identified [24]. *P. notoginseng* is used as a functional supplement, as well as an industrial material for saponins production [25]. Recently, the genome of *P. notoginseng* has been sequenced and published, providing insights into the functional identification of genes involved in various processes [12,26]. To date, a few transcriptomes of *Araliaceae* have also been published [27,28]. However, most of the previous works focused on secondary metabolism, and the sequence data are still insufficient for functional studies on genes associated with desiccation sensitivity. Significantly reduced water contents and survival ratios of *P. notoginseng* seeds during the desiccation process indicated that recalcitrant *P. notoginseng* seeds could only be stored at room temperature for several days (Figure 1). It would be valuable to identify the underlying control of seed desiccation tolerance so that crop improvement could prolong the storage life of *P. notoginseng* seeds. In the present study, transcriptomes from control (0 h) and desiccated embryos (36 h) were used to identify the genes associated with the desiccation sensitivity of *P. notoginseng*.

Desiccation tolerance is present in many resurrection plants and seeds [29]. The basic metabolism, including glycolysis and TCA cycle, provides most of the energy for seed development. Metabolites in carbohydrate metabolism have been identified as one of the most important contributors to desiccation tolerance [30]. Sugar accumulation occurs when plants encounter dehydration, and the correlation of sugar accumulation and desiccation tolerance has been well studied [31]. For example, raffinose and stachyose protect plant cells from oxidative damage caused by dehydrated conditions [32]; trehalose has been reported to be the main factor in the acquisition of desiccation tolerance in Selaginella species [33]. And octulose accumulation has been considered to be correlated with desiccation tolerance in desiccation-tolerant species [34]. In *P. notoginseng*, the expression of most glycolysis-related genes changes significantly during the desiccation process, suggesting a potential role of glycolytic flux in desiccation tolerance. In desiccation-tolerant seaweed species, the relative mRNA levels of genes associated with basal metabolism, such as the *pyruvate dehydrogenase (PDH)* gene, were over-expressed during the desiccation process [35]. In our study, the expression of most TCA cycle-related genes was reduced. For example, the transcript levels of two *PDH* genes in *P. notoginseng* decreased more than two-fold at SQS36 compared with SQS0. The differential expression of central metabolism (glycolysis and TCA) genes may be correlated with the desiccation sensitivity of *P. notoginseng* seeds.

Desiccation leads to different physiological and transcriptional responses in plants. In *P. notoginseng*, enrichment analysis highlighted a KEGG term, ‘Plant hormone signal transduction’, which consisted of the largest number of DEGs. A number of DEGs involved in various hormones, including auxin, cytokinin, jasmonic acid, ABA, ethylene, brassinosteroid, and salicylic acid, were identified, indicating that the desiccation sensitivity of *P. notoginseng* may be controlled by an intricate hormonal signaling network [36], which is similar to the recalcitrant seeds of *Quercus variabilis* [37]. Among these hormones, the roles of hormone ABA in the response to desiccation in tolerant plants have been widely studied [38]. The application of exogenous ABA dramatically strengthens the ability to survive in dehydrated conditions [39]. Increasing evidence has suggested a conserved regulatory machinery of ABA-mediated gene expression for desiccation tolerance in various recalcitrant seeds [40]. The phytohormone ABA was recognized in a family of *PYR/PYL* receptors, identified by pyrabactin, a synthetic inhibitor of seed germination, in various plants [41]. In *P. notoginseng*, the expression of four ABA receptor *PYR/PYL* family genes was down-regulated by desiccation in natural conditions. Reduced ABA sensitivity may be correlated with the phenomenon of seed recalcitrance in *P. notoginseng*.

Lipid changes mainly occur in dehydration-sensitive plants under drought stress [42]. The comparison of lipid composition between the desiccation-tolerant plant and desiccation-sensitive plant suggests that lipids, such as phosphatidylinositol, are involved in the acquisition of desiccation tolerance [11]. In our study, the expression of a large number of fatty acid-related genes was changed during the desiccation process in *P. notoginseng*. Interestingly, most of the fatty acid biosynthesis- and elongation-related genes were reduced, and more than half of the fatty acid degradation-related genes were induced at SQS36 compared with SQS0. Altered fatty acid contents may lead to the instability of membranes and proteins.

LEA proteins, protective molecules against desiccation stress, are reported to be involved in the replacement of water, sequestering ions, and removing ROS under dehydrated conditions [21,40]. During the germination of seeds, the abundance of several *M. truncatula* LEA proteins was up-regulated, and a sugar beet EM-like protein GEA1 was down-regulated [43,44]. It has been reported that six LEA proteins (EM1, EM6, MP2, PM25, LEAm, and SBP65) accumulated only at low levels, and six (PM1, D113.I, 2 D34 members, PM10, and PM18) were undetectable in recalcitrant *Castanospermum australe* seed proteome [20]. In *P. notoginseng* embryos, 53 *LEA* genes were identified, of which 14 genes showed significant changes, 9 up-regulated and 5 down-regulated, providing a great deal of candidate genes associated with desiccation sensitivity.

Dehydration disrupts the normal metabolisms of seeds and leads to the accumulation of ROS, which can damage cellular components at a high concentration [45]. The level of ROS should be strictly regulated in the cell. Thus, the removal of ROS, such as H_2_O_2_ and O^2−^, contributes to seed desiccation tolerance [22]. H_2_O_2_ scavenging is mainly accomplished by the ascorbate-glutathione cycle via several coupled redox reactions involving several enzymes, including MDHAR, DHAR, and GR [46]. In our study, genes encoding MDHAR, DHAR, and GR were identified, and most of these genes showed differential expressions during the desiccation process in *P. notoginseng*. In Arabidopsis, three MDHARs were highly accumulated during histodifferentiation [47]. In *P. notoginseng*, one MDHAR (c73699_g1), one GR (c76376_g3), and one DHAR (c73699_g1) were induced at SQS36 compared with SQS0. This is similar to recalcitrant tea seeds (*Camellia sinensis* L.) [48]. The changes in antioxidant enzymes may play an important role in desiccation sensitivity.

In addition to the ascorbate-glutathione cycle, some other antioxidant enzymes participate in reserve deposition during maturation drying. In rice, the contents of several thiol-dependent antioxidant enzymes, such as thioredoxin peroxidases (TPX) and glutathione peroxidases (GPX), increased during reserve deposition [49]. In contrast, in *P. notoginseng* embryos, two *TPX* genes decreased significantly in abundance during the desiccation process, suggesting that the reduction in thiol-dependent antioxidant enzymes may be involved in desiccation sensitivity. The detoxification of aldehydes is another essential step for seed development. For example, an important role of *ADH* for seed development has been observed in several orthodox seeds, such as rice seeds and maize seeds [49]. In *P. notoginseng* seeds, a number of *ADH* genes were identified as DEGs, indicating the occurrence of complicated changes in the regulation of *ADH* accumulation during the desiccation process of *P. notoginseng* seeds.

## 4. Materials and Methods

### 4.1. Plant Material, Treatment, and Sampling

*P. notoginseng* (Burk.) F. H. Chen berries were harvested from 3-year-old plants in mid-November, 2015, in Wenshan Institute of Sanchi Ginseng, Wenshan, China. Seeds were removed from the berries by washing under water. The seeds were sterilized with 5% sodium hypochlorite and surface-dried under air condition. Seeds were mixed with moist perlite (2.2 g·g^−1^) (seeds/perlite = 1:3, *v*/*v*) and stratified at 10 °C in darkness for 25 d until the embryos fully developed. After stratification, all the seeds were washed and surfaced-dried under air conditions. Then, the seeds were divided into 2 groups and dehydrated in open containers in a controlled environment room [20 °C, 55% relative humidity (RH)] for 0 h and 36 h. Three replicates of 25 embryos excised from the seeds for each group were dipped quickly in liquid N_2_ and then stored at −80 °C for RNA extraction. Three replicates of 50 seeds for each group were germinated in a 9 cm plastic culture dish filled with moist perlite at 20 °C in darkness. Three replicates of 20 seeds and embryos for each group were used for the determination of water content.

### 4.2. RNA Isolation and Library Construction

Total RNAs were isolated from *P. notoginseng* seed embryos using a TRIzol Kit (Promega, Beijing, China) according to its protocol and quantified using a Bioanalyzer 2100 (Agilent, Beijing, China). The quality of RNA was monitored using an RNA 6000 Nano LabChip Kit (Agilent, Santa Clara, CA, USA). A total of 10 μg of RNA was prepared for the construction of cDNA libraries. RNA was subjected to oligo-dT-attached magnetic beads (ThermoFisher, Shanghai, China) and fragmented into small fragments. The synthesized cDNA fragments were purified and ligated to Illumina adapters. The ligation products were fractioned and excised for PCR amplification. The amplified fragments were sequenced on an Illumina HiSeq™ 2500 platform (Gene Denovo Co., Guangzhou, China).

### 4.3. Sequence Assembly and Functional Annotation

Before the de novo assembly, low-quality reads, including the reads containing low Q-value bases (>20%), the reads with unknown bases (>5%), and the reads containing adaptor sequences, were excluded. The clean reads were assembled into unique consensus sequences using Trinity v2.0.6 [50]. All unigenes were searched against various protein databases, including the NCBI nr protein database, Swiss-Prot protein database, Kyoto Encyclopedia of Genes and Genomes (KEGG) pathway database, and KOG database using a BLASTX alignment algorithm with an E value < 0.0001. The Blast2GO program was applied to generate the Gene Ontology (GO) annotation for each unigene. Metabolic pathway annotation was carried out using the BLASTall program against the KEGG online database.

### 4.4. Differentially Expressed Genes (DEGs) Screening

All the reads representing each unigene were mapped to the assembled transcripts by the ‘single-end’ method with parameter ‘-v 3 -a–phred64-quals’ through the alignment software Bowtie v0.12.8. To calculate the unigene expression, the mapped reads representing each unigene were numbered and normalized into a Reads Per Kb per Million reads (RPKM) value. Significant DEGs were identified under the threshold of a false discovery rate (FDR) < 0.001 and an |log2(change) ratio| > 1 using the edgeR package [51]. Enrichment analyses of the DEGs in GO and KEGG terms were carried out according to a reported method [52].

### 4.5. PPI Analysis

For PPI prediction, amino acid sequences of the DEG encoding proteins were searched against the STRING database ver. 10.5. Only the PPI interactions between proteins from the searching data group were selected, and the confidence score was set to be ≥0.7 (high confidence). The results of the PPIs were visualized using Cytoscape software Ver., 3.2.1.

### 4.6. Statistical Analyses

Differences in values between the different groups were analyzed using a one-way analysis of variance with Student’s *t*-test at a significance level lower than 0.05. For the experiments, three biological repeats were performed, and the values shown in the figures represent the average values of the three repeats.

### 4.7. Data Validation via Quantitative Real-Time PCR (qPCR)

To verify the accuracy of the transcriptomic sequencing data, twelve DEGs were selected for qPCR analysis. Total RNA was from the same samples as the above-mentioned library construction. RNA samples were analyzed in biological triplicate and technical triplicate for qPCR. Quantitative PCR was performed using StepOnePlus™ (TMO.US, MA, USA) and the SYBRPremix Ex TaqTM (TliRNaseH Plus) Kit (TaKaRa, Kyoto, Japan, Code No. RR420A) according to the manufacturer’s protocol. Gene-specific primers of sixteen target genes and one housekeeping gene (18 s) were designed using Oligo 6.0 software and synthesized by Shanghai Langjing Biotechnology Co., Ltd. (Shanghai, China) The primer sequences are listed in Table S10. The 20 μL PCR mixture consisted of 10 μL SYBR^®^ Premix Ex Taq (TliRNaseH Plus), 0.4 μL PCR Forward Primer, 0.4 μL PCR Reverse Primer, 0.4 μL ROX Reference Dye (50×), 2 μL DNA template, and RNase-free water to a total volume of 20 μL. The qPCRs were performed with the following conditions: denaturation at 95 °C for 30 s, 40 cycles of denaturation at 95 °C for 5 s, annealing at 60 °C for 30 s, and extension at 72 °C for 30 s. Relative gene expression was calculated using the 2^−ΔΔCt^ method [53]. Excel 2010 was used to calculate the correlation coefficient between the quantitative expression using qPCR and transcriptome analysis.

## 5. Conclusions

In our study, we explored the transcriptomic changes in *P. notoginseng* embryos during a desiccation process. Two independent groups of cDNA libraries from control (0 h) and dehydrated seeds (36 h) of *P. notoginseng* were separately sequenced. A great number of DEGs were identified during the desiccation process. The expression of the genes involved in central metabolism, hormone signaling, fatty acid metabolism, and ascorbate-glutathione cycle showed significant changes during the desiccation process. Our data provide a comprehensive resource to identify genes which could potentially improve the desiccation tolerance of *P. notoginseng* seeds.

## Figures and Tables

**Table 1:** Identification of genes associated with removal of ROS.

Removal of ROS-Related Cycle	Gene_id	Description	log2ratio	*p* Value
Thioredoxin peroxidase	c74134_g1	thioredoxin peroxidase	−2.3605	0.025133
Aldehyde dehydrogenase	c68124_g2	aldehyde dehydrogenase F1-like	−1.9587	2.24 × 10^−19^
	c66461_g1	aldehyde dehydrogenase	−1.1273	8.10 × 10^−10^
	c68124_g1	aldehyde dehydrogenase F1-like isoform X2	−1.2546	1.31 × 10^−8^
	c72774_g1	aldehyde dehydrogenase family 7 member B4	0.99753	3.64 × 10^−7^
	c63342_g2	Aldehyde dehydrogenase	−1.0448	1.04 × 10^−5^
	c72774_g3	aldehyde dehydrogenase isoform X1	−2.6146	0.0068078
	c66461_g4	aldehyde dehydrogenase	−2.2715	0.010229
Ascorbate-glutathione	c73699_g1	monodehydroascorbate reductase 1	1.1042	3.31 × 10^−9^
	c75404_g1	glutathione reductase	−1.3046	1.62 × 10^−8^
	c73699_g1	dehydroascorbate reductase	1.1042	3.31 × 10^−9^
	c77787_g3	dehydroascorbate reductase	−1.7284	2.64 × 10^−10^

## Data Availability

The sequencing data from this study have been submitted to the NCBI Sequence Read Archive (SRA) (https://www.ncbi.nlm.nih.gov/sra, accessed on 30 September 2025) under the accession number PRJNA794283.

## Supplementary Materials

The following supporting information can be downloaded at: https://www.mdpi.com/article/10.3390/plants12223881/s1, Figure S1: sequencing, assembly and functional annotation of transcripts; Figure S2: protein-protein interaction (PPI) network analysis of DEGs; Table S1: the detial information of raw Illumina sequencing reads; Table S2: the detail information of 56 functional GO terms; Table S3: the detail of KEGG signaling and metabolic pathways; Table S4: the detail information of 12025 DEGs; Table S5: the detial information of 28 enriched GO terms; Table S6: the detail information of 310 enriched KEGG pathways; Table S7: the detail information of 17 ABA signaling genes; Table S8: the expression pattern of the genes related to fatty acid-related KEGG terms; Table S9: identification of LEA protein encoding genes; Table S10: gene primers of qPCR.
