# Supplementary material for: Analysis and Identification of Genes Associated with the Desiccation Sensitivity of Panax notoginseng Seeds
Source: Plants (Basel). 2023 Nov 17;12(22):3881. doi: 10.3390/plants12223881 (PMC10674602; doi:10.3390/plants12223881)
Supplement: Supplementary file 1 [file plants-12-03881-s001.zip › Table S10.pdf]

Table S10. gene primers of qPCR.

Number	Gene ID	Primer	For（5’-3’）	Length(bp)
1	1-c72904_g1-1F	F	GCATTGGACCTTGGTGGAG	19
	1-c72904_g1-1R	R	CCTACCAGGAGGAAGATGAAA	21
2	2-c71080_g5-1F	F	TAAGAAGATTATTGCCGTGCT	21
	2-c71080_g5-1R	R	CGATACATACCTAACCTCACCAGT	24
3	3-c64491_g1-1F	F	TGAGAAAACGATACAACAGAGATT	24
	3-c64491_g1-1R	R	GCTACTTGTTCTCCCTCCTTCA	22
4	4-c72192_g1-1F	F	GACCCTACAGAGCAAACCAAA	21
	4-c72192_g1-1R	R	AACAGCAAGTGATACTGCCAAG	22
5	8-c66826_g1-1F	F	TTGTTGAGTCCTCCCTCCGA	20
	8-c66826_g1-1R	R	GACTTCCAGAGCCTGTTGTTCA	22
6	10-c71182_g3-1F	F	ATCGCTTCTTCATTCTTTGGC	21
	10-c71182_g3-1R	R	ATGGTGACCGCATTCGTG	18
7	11-c42049_g2-1F	F	AGGCGGGAATGTAGGCAATC	20
	11-c42049_g2-1R	R	GCTCGGACTATCTCGGCG	18
8	12-c63527_g1-1F	F	AACAAGGCAGGGCAGAGG	18
	12-c63527_g1-1R	R	AGCATGTCACGAGCCAAGTC	20
9	13-c72811_g2-1F	F	GGTTATGGAAAGATGTCCTGATT	23
	13-c72811_g2-1R	R	TTCTCCCTCCAGTCTTAAATGC	22
10	14-c75404_g1-1F	F	CGATAGCACGGTGGGAATACA	21
	14-c75404_g1-1R	R	AATCCGCCTCGTTACTGACC	20
11	15-c66673_g1-1F	F	CAAGAAAGTTGAGGCAGCAGT	21
	15-c66673_g1-1R	R	GTTGAATAGGTGGGAATGTCG	21
12	16-c76376_g3-1F	F	ATTGTGGACCCGCATACAGT	21
	16-c76376_g3-1R	R	GCAAATCAAGGGCAGCATC	21
13	18S rRNA-F	F	GATGCGCTCCTGTCCTTAAC	20
	18S rRNA-R	R	CATCCTTGGCAAATGCTTTC	20
