# Supplementary figures and images for: Analysis and Identification of Genes Associated with the Desiccation Sensitivity of Panax notoginseng Seeds
Source: Plants (Basel). 2023 Nov 17;12(22):3881. doi: 10.3390/plants12223881 (PMC10674602; doi:10.3390/plants12223881)

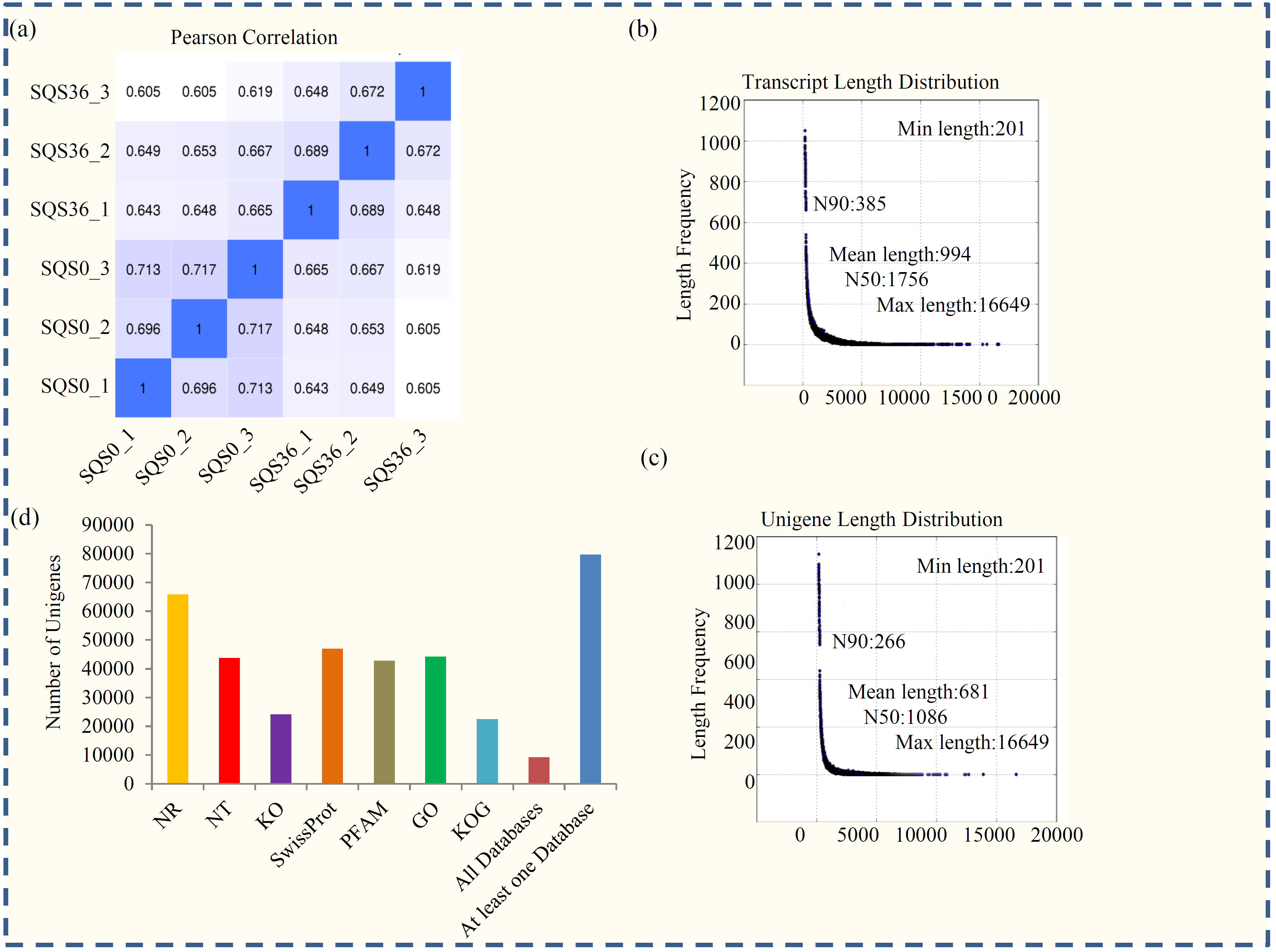

Supplement: Supplementary file 1 [file plants-12-03881-s001.zip › Figure S1 sequencing, assembly and functional annotation of transcripts.tif]

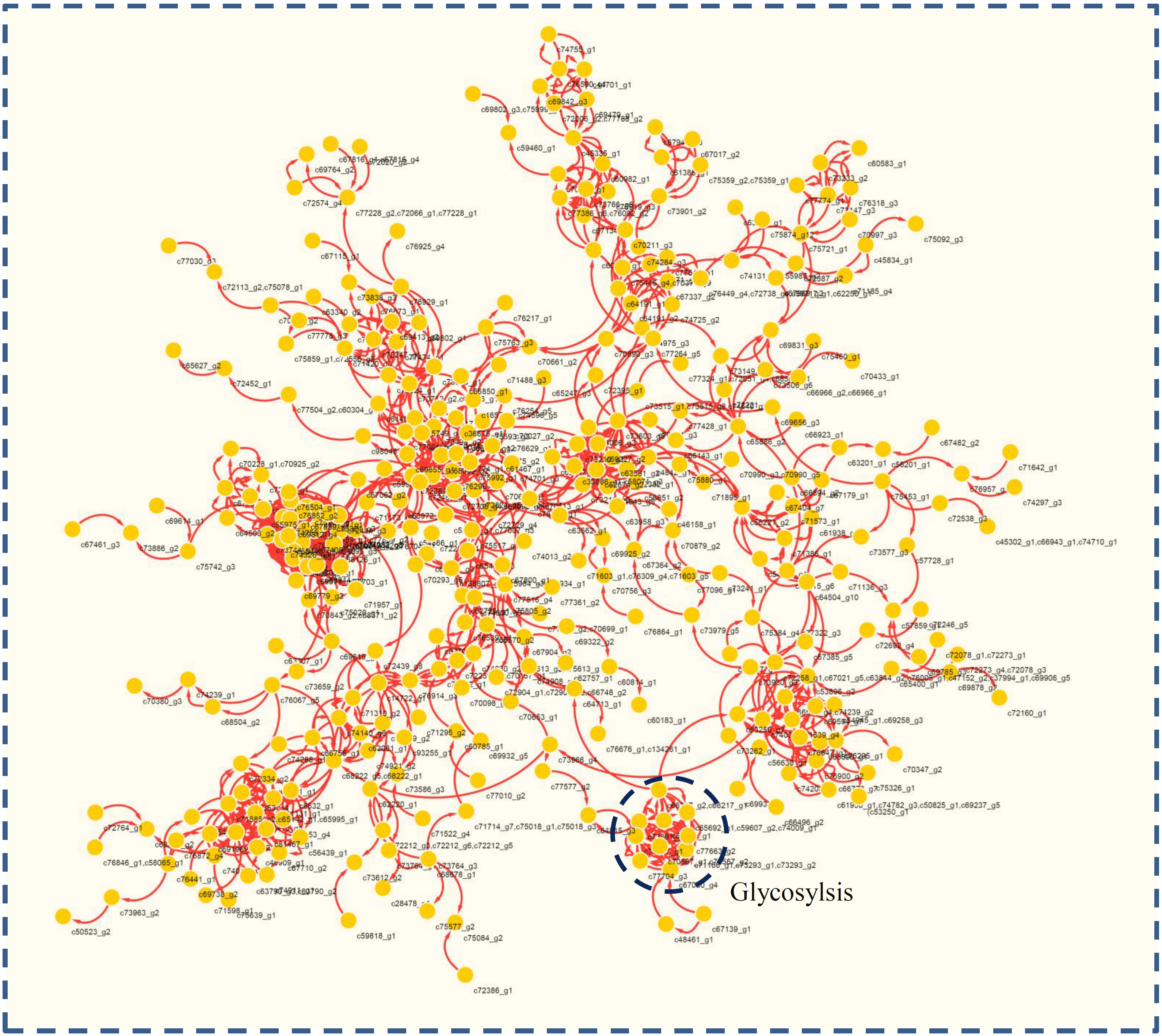

Supplement: Supplementary file 1 [file plants-12-03881-s001.zip › Figure S2 protein-protein interaction (PPI) network analysis of DEGs.tif]
